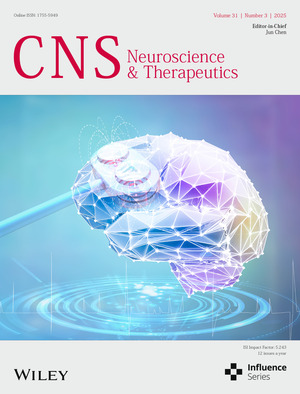# Front Cover

**DOI:** 10.1111/cns.70363

**Published:** 2025-04-01

## Abstract

Cover image: The cover image is based on the article *High‐Frequency rTMS Improves Visual Working Memory in Patients With aMCI: A Cognitive Neural Mechanism Study* by Yun‐Xia Li et al., https://doi.org/10.1111/cns.70301.